# An overview of microbial indigo-forming enzymes

**DOI:** 10.1007/s00253-019-10292-5

**Published:** 2019-12-13

**Authors:** Andrea N. Fabara, Marco W. Fraaije

**Affiliations:** Molecular Enzymology group, University of Groningen, Nijenborgh 4, 9747 AG Groningen, The Netherlands

**Keywords:** Indigo, Indole, Naphthalene dioxygenase, Styrene monoxygenase, P450 monoxygenase, Peroxygenase, Flavoprotein monooxygenase

## Abstract

Indigo is one of the oldest textile dyes and was originally prepared from plant material. Nowadays, indigo is chemically synthesized at a large scale to satisfy the demand for dyeing jeans. The current indigo production processes are based on fossil feedstocks; therefore, it is highly attractive to develop a more sustainable and environmentally friendly biotechnological process for the production of this popular dye. In the past decades, a number of natural and engineered enzymes have been identified that can be used for the synthesis of indigo. This mini-review provides an overview of the various microbial enzymes which are able to produce indigo and discusses the advantages and disadvantages of each biocatalytic system.

## Introduction

**Fig. 1:**
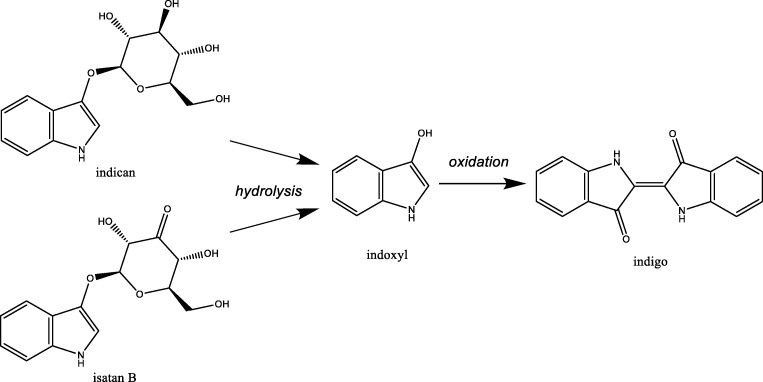
Structural formulas of indigo, indoxyl, and some of its plant precursors, indican and isatan B

Humans have decorated textiles with dyes and pigments, often derived from plant material, since ancient times (Aino et al. 2018). Indigo blue (in short: indigo) is one of the oldest dyes to be used for textile dyeing (Fig. 1). In 2009 at Huaca Prieta, Peru, the oldest indigo-dyed cotton fabric was discovered, dating back to 6000 years ago (Splitstoser et al. 2016). Indigo-dying was also known in ancient Egypt; the funerary wardrobe of Tutankhamun included an indigo-dyed state robe. Clearly, the use of indigo as dye was widespread already early in human history (Clark et al. 1993).

Traditionally, indigo has been prepared from various plants. This is done by fermenting plant material, which will liberate indoxyl from indigo precursors, typically glycosylated forms of indoxyl such as indican and isatan B (Fig. 1). Through air-mediated oxidation, indoxyl then spontaneously dimerizes to form indigo. To dye textiles, indigo is first chemically reduced which results in the soluble reduced form of indigo, leucoindigo. The indigo-dying process, in which indigo is chemically treated, was traditionally done in buckets or vats, hence the classification as a vat-dye. The deposition of the insoluble indigo on the fabric results in a special decoration of the fabric material as the fabric is not penetrated by the dye. This gives denim and other indigo-dyed garments their special appearance, where wear or abrasion exposes the white interior of the fiber.

The most effective plant-based process of producing the indigo dye involved the plant *Indigofera tinctoria*, which is distributed throughout the tropical and subtropical regions of the world and particularly in India. In fact, the Greeks referred to the dye as “indikon” which means “from India.” Yet, also in more moderate climate regions, indigo has been produced and used from alternative plants. In Europe, woad (*Isatis tinctoria*) was traditionally used as the source of indigo. In fact, for centuries, there were many regions where woad cultivation and processing was a dominant industry. However, through increasing trade between Europe and other continents, the import of indigo from India fully eradicated woad-based industries starting in the 16th century. While *Indigofera* contains relatively high concentration of indican, woad contains significantly lower amounts of two indigo precursors: indican and isatan B (Fig. 1) (Gilbert et al. 2004).

At the advent of the 20th century, a petroleum-based chemical process to synthesize indigo was introduced. This rapidly evolved into an effective industrial process and replaced most of the plant-based production of indigo.

## Chemical production of indigo

**Scheme 1:**
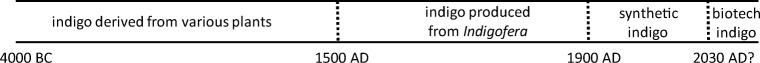
Timeline of indigo production processes

About 150 years ago, Adolf von Baeyer succeeded in elucidating the structural formula of indigo. He was also the first in developing a method for the chemical synthesis of indigo; in 1870, he described the synthesis of indigo starting from isatin. In subsequent years, Baeyer and others developed alternative synthetic routes for the synthesis of indigo, but most of them started from expensive starting material and were not commercially viable. Yet, within a few decades, several effective indigo production processes were developed (Scheme 1), and indigo production at tonne scale was established. Industrial production of indigo started around 1900 and developed into the current large scale industrial processes, replacing virtually all former plant-based production sites. Current large scale production processes typically rely on aniline as a cheap fossil-based feedstock.

## Alternative indigo production processes

Indigo has been the world’s most important and popular dyestuff, and the demand is still increasing, especially in the denim industry. Plant material, mainly leaves, contains indican as a colorless compound. By fermentation, indican is hydrolysed into β-D-glucose and indoxyl, while in a next step, using air as oxidant, indigo is obtained as solid material (Fig. 1). Current chemical production processes do not start from renewable material but instead depend on relatively cheap petrochemical precursors. The chemistry used for the industrial production of indigo is quite harsh, not environmentally friendly, and leads to problems in the disposal of the dye waste. Because these factors are not priced in, this is a commercially viable process. To prevent the formation of large waste streams and to be able to use a renewable feedstock, it is desirable to develop a more sustainable process. For this, biotechnological approaches are being explored.

Indigo production based on plant material, reviving and optimizing the traditional plant-based processes, could be an option. This would involve the breeding of plants with high indican content and other beneficial traits. Recently, obtained genetic insights in the natural indigo synthesis pathway in plants could be exploited for this (Jin et al. 2016; Inoue et al. 2017), and it has already been shown that through genetic engineering, plants can be bred that produce various indigo dyes (Fräbel et al. 2018). However, production of indigo using plants would compete with food and feed production.

As an alternative and promising approach, the production of indigo by fermentation has been explored since the early 1980’s when the first bacterial strains capable of indigo synthesis were identified. Among the discovered microbial indigo producers, the majority are aromatic hydrocarbon-degrading bacteria (Bhushan et al. 2000). Although various bacterial strains and enzymes have been identified to be able to produce indigo, there is still no large-scale industrial biotechnological process for producing indigo.

With the current realization that production processes should be (more) sustainable, the demand for a biotechnological indigo production process becomes more urgent, and modern techniques for enzyme and strain engineering make it more feasible. In the next paragraphs, details of the microbial enzymes known to form indigo are discussed. In all cases, these enzymes oxidize indole to form indoxyl, which spontaneously dimerizes into indigo. Besides naturally occurring enzymes, enzymes engineered towards indigo production will also be discussed.

## Microbial enzymes producing indigo

In the last few decades, a large number of indigo-producing enzymes have been identified in microorganisms, mainly in bacteria. In the past, indigo production was observed when studying a particular bacterial strain, and in recent years, a number of indigo-producing enzymes were discovered through metagenome mining (Lim et al. 2005; van Hellemond et al. 2007; Singleton et al. 2012; Nagayama et al. 2015; Ma et al. 2018). The discovery of such enzymes was often a serendipitous finding, based on the pink or bluish appearance of colonies or cultures. All these enzymes convert indole through an oxygenation reaction. In most cases, this is catalyzed by using electron donating cofactors and molecular oxygen, but also peroxide-driven peroxygenases have been shown to convert indole into indigo. The identified redox enzymes can be dissected into three different enzyme classes based on the cofactor used by the enzymes to perform the oxygenation of indole: non-heme iron oxygenases, heme-containing oxygenases, and flavin-dependent monooxygenases.

## Non-heme iron oxygenases

### Naphthalene dioxygenases

**Fig. 2:**
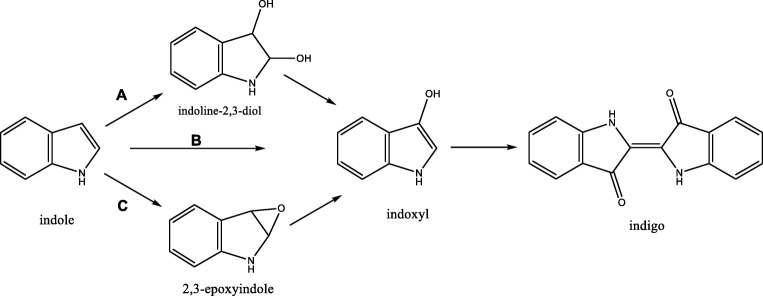
Different enzymatic routes towards indigo, either via dioxygenation (**A**), direct hydroxylation to indoxyl (**B**), or via epoxidation (C)

The realization that microbial enzymes can be used for indigo production started in 1983 when studying naphthalene dioxygenase-encoding genes from *Pseudomonas putida* G7. The corresponding multi-component naphthalene dioxygenase (NDO) system was expressed in *Escherichia coli* resulting in blue-colored cultures (Ensley et al. 1983). Indigo production could be boosted by adding tryptophan or indole to the medium. This suggested that the oxygenase was capable to convert indole into indoxyl, leading to indigo formation. Yet, the initial (labile) product of action of NDO is indoline-2,3-diol which quickly decays to form indoxyl, which subsequently condenses into indigo (route A in Fig. 2).

NDO (EC 1.14.13.8) has a broad substrate range and catalyzes several types of reactions including *cis*-dihydroxylation, monooxygenation, and desaturation reactions (Parales et al. 2000). Building on this finding, through a combination of tuning expression levels, protein engineering, and optimizing medium composition, it was shown that a recombinant *E. coli* could be used to produce indigo from glucose (Murdock et al. 1993). While the productivity (135 mg/L) was still modest, this demonstrated that a fermentative approach can be effective. Since then, many other aromatic hydrocarbon-degrading bacteria have been identified and studied for their capability to produce indigo, often with indole as precursor (O’Connor and Hartmans 1998; Bhushan et al. 2000; Alemayehu et al. 2004; Pathak and Madamwar 2010; Qu et al. 2012b). While many of these bacteria are pseudomonads, other bacteria have also been found to harbor NDOs that can be used to produce indigo (Mercadal et al. 2010; Qu et al. 2010).

NDO is a multi-component enzyme system that allows the respective organism to hydroxylate naphthalene and related compounds to facilitate full metabolism of such aromatic compounds. Besides the oxygenase enzyme component, it also involves a flavin-dependent reductase and a ferredoxin component to generate the electrons required by the oxygenase. Details on how NDO performs catalysis were revealed when its crystal structure was elucidated (Kauppi et al. 1998). The oxygenase forms an α_3_ß_3_ heterohexameric structure composed of two different subunits, the α- and ß-subunits. The active site resides in the α-subunits and contains a non-heme iron cofactor. The iron is liganded by two histidines and an aspartate. The active site is rather buried, connected with a tunnel to the protein surface for substrate/product diffusion. Electrons from ferredoxin are delivered to the iron via a Rieske [2Fe–2S] center located in the neighboring α-subunit at a distance of 12 Å. The tunnel and shape of the active site determine the substrate specificity of NDO and sequence-related oxygenases. A number of NDO homologs have been identified that are similar in sequence but vary significantly in substrate scope. These include toluene dioxygenases, cumene dioxygenases, and biphenyl dioxygenases (Groeneveld et al. 2016). Overlap of substrate profiles has been observed as some cumene, and toluene dioxygenases were shown to produce indigo from indole (Stephens et al. 1989; Woo et al. 2000; Groeneveld et al. 2016).

Recombinant *E. coli* strains expressing NDO have been reported to produce 36–300 mg indigo per liter of culture broth. These studies have shown that for an optimal production of indigo, tuning of expression and growth conditions is crucial. Besides for adding tryptophan, indole and/or glucose and other medium components can also affect productivity. The addition of iron to the medium was suggested to boost the functioning of the coexpressed ferredoxin (Murdock et al. 1993). This also hints to the complexity of the NDO system for functional expression, 4 genes need to be coexpressed. With multiple enzyme components and the requirement of incorporation of Fe-S clusters and a catalytic iron, the NDO system may not be the ideal biocatalytic tool.

### Multicomponent phenol hydroxylases

Another multi-component enzyme class that was shown to be capable to produce indigo are the bacterial multicomponent phenol hydroxylases (mPHs) (EC 1.14.13.7). mPHs consist of a hexameric hydroxylase component composed of three different subunits (αβγ)_2_: a flavoprotein reductase to generate and deliver electron to the oxygenase component and a cofactorless regulatory protein (Nordlund et al. 1990; Leahy et al. 2003). For catalysis, the α-oxygenase subunit contains a carboxylate-bridged diiron center. The mPHs share features with the soluble methane monooxygenases. Being effective in mono-hydroxylating phenol, it is likely that indigo formation is achieved through hydroxylation of indole into indoxyl (route B in Fig. 2). A related multicomponent diiron enzyme system has also been reported to be able to produce indigo, toluene-4-monooxygenase (Yen et al. 1991). Engineering of this bacterial monooxygenase resulted in mutants with different regioselectivities when hydroxylating indole, thereby producing different indigoids (McClay et al. 2005). Only a few reports on the use of mPHs for indigo production have appeared (Qu et al. 2012a). This may partly be due to the fact that these types of oxygenases have been discovered quite recently. The reported studies on using mPH-harboring microbes for indigo production suggest that these multi-component enzymes can indeed be used in recombinant hosts. Expression in *E. coli* led to a production of 52 mg indigo per liter of medium (Doukyu et al. 2003). However, it is worth noting that the original strain from which the genes were cloned, *Acinetobacter* sp. ST-550, reached higher titers of indigo (292 mg/L) (Doukyu et al. 2002). Similar to the NDOs, it may be challenging to find optimal conditions for functional and stable expression of all components of a mPH in a recombinant host.

## Heme-containing oxygenases

### Cytochrome P450 monooxygenases

Cytochrome P450 monooxygenases (CYPs) (EC 1.14.14.X) contain a heme as redox cofactor, enabling catalysis of various oxidation reactions. A plethora of CYPs exist in nature with highly diverse physiological roles. Microbes often employ CYPs in catabolic pathways. A thoroughly studied CYP is the P450 BM3 (CYP102A1), a fatty acid hydroxylating CYP from *Bacillus megaterium* ATCC 14581. This CYP is attractive as biocatalyst because it contains a flavin-containing reductase domain in addition to the typical P450 monooxygenase domain. The reductase domain renders the CYP self-sufficient; it does not rely on a secondary reductase but merely requires NADPH to function. Furthermore, P450 BM3 can be easily expressed in *E. coli* and is rather stable. The wild-type enzyme is not capable to convert indole. Yet, in 2000, directed evolution of P450 BM3 accidentally resulted in mutant enzymes that produced indigo (Li et al. 2000). By introducing three mutations, a variant was created which displays a high activity on indole (*k*_cat_ = 2.7 s^−1^(Li et al. 2000a)). Around the same time, it was discovered that also some human CYPs convert indole into indigo (Gillam et al. 1999; Gillam et al. 2000; Banoglu et al. 2001). Conversion by CYPs was found to proceed via formation of indoxyl (Gillam et al. 2000) (route B in Fig. 2). However, as eukaryotic CYPs are typically rather slow in catalysis, human or other mammalian CYPs are enzymes not suitable for a biotechnological process. For this, engineered variants of P450 BM3 or other microbial CYPs are more suitable. A recent study has demonstrated that the performance on indole of the above-mentioned triple mutant of P450 BM3 could be improved by one order of magnitude through directed evolution. The improvement was brought about by merely adding one more mutation. Moreover, in the last two decades, more microbial CYPs have been reported for their capacity of indigo formation (Brixius-Anderko et al. 2017; Fiorentini et al. 2018; Kim et al. 2018) which may also represent good starting points for creating an optimized CYP for indigo production. The feature that conversion of indole into indigo can be seen by eye makes it attractive to apply directed evolution protocols. Such colorimetric detection allows to screen large mutant enzyme libraries.

### Unspecific peroxygenases

The oxygenases described above all depend on reduced coenzymes, such as NADH and NADPH, as electron donors. One class of enzymes that would eliminate such cofactor dependency is the unspecific peroxygenases (UPOs) (EC 1.11.2.1). These redox enzymes are capable of oxygenations with the help of hydrogen peroxide. Similar to CYPs, UPOs contain a tightly bound heme cofactor that catalyzes the oxygenations. However, in UPOs, the required oxygen and electrons for an oxygenation reaction are delivered indirectly, by using hydrogen peroxide as substrate. Recently, it was described in a patent that an UPO can convert indole into indigo (Kalum et al. 2014; Martínez et al. 2017) by using hydrogen peroxide as oxidant. Besides for indole, the studied fungal peroxygenase can also be used for the preparation of other indigoids. The reaction proceeds via formation of 2,3-epoxyindole as intermediate product (route C in Fig. 2). This rearranges spontaneously into indoxyl but also the side-product 2-oxindole is formed. This mixture of products prevents full conversion of indole into indigo. So far, UPOs have only been identified in fungi (Faiza et al. 2019). The recombinant production of UPOs has been found to be problematic which has hampered thorough biochemical investigations, enzyme engineering studies, and applications of these redox enzymes. Effective expression systems will need to be developed before UPOs can be considered for large scale industrial processes. Another weakness of UPOs is that they can easily become inactivated by hydrogen peroxide. Careful dosing of hydrogen peroxide is required for optimal usage of the biocatalyst. Enzyme engineering may also help in generating variants that are less sensitive towards inactivation.

Other heme-containing enzymes might offer another approach in developing peroxygenases for indigo production. It has been reported that the marine dehaloperoxidase-hemoglobin from *Amphitrite ornata* and the mammalian heme-containing indoleamine 2,3-dioxygenase can act as peroxygenases when offered indoles and hydrogen peroxide (Kuo and Mauk 2012; Barrios et al. 2014). However, it seems that these enzymes are rather slow and also generate other indole-based products. Moreover, their mammalian origin makes them hard to use in microbial fermentations. Inspired by the catalytic power of heme-dependent enzymes, chemical approaches are also explored in which bio-inspired catalysts are designed, based on the iron-porphyrin structure of heme cofactors (Rebelo et al. 2014). This is a new approach to produce indigo based on a simple and cost-effective model system of the enzymes. The method considers the oxidation of indole by hydrogen peroxide by using an iron(III)porphyrin in ethanol as solvent. The yields of indigo depend on the type of metalloporphyrin system used and on the control of the oxidation conditions.

## Flavin-dependent monooxygenases

Several flavin-dependent monooxygenases have been reported to be able to produce indigo from indole. All these indigo-producing enzymes belong to four distinct flavoprotein monooxygenase classes (van Berkel et al. 2006) and are discussed below.

### Styrene/indole monooxygenases

The FAD-dependent styrene monooxygenases (EC 1.14.14.11), belonging to the class E flavoprotein monooxygenases (van Berkel et al. 2006), are two-component systems which rely on a flavin reductase and a monooxygenase. In most cases, these two components are encoded by separate genes, but in a few cases, the components are fused (van Hellemond et al. 2007; Tischler et al. 2009). The reductase uses NADH to generate reduced FAD, which subsequently has to find the monooxygenase by diffusion. The monooxygenase is able to form a reactive peroxyflavin intermediate upon binding reduced FAD by reacting with dioxygen, which subsequently can react with styrene. Styrene monooxygenases are typically highly enantioselective, which makes them attractive biocatalysts. Styrene monooxygenases represent also one of the few flavoprotein monooxygenases capable of performing epoxidations. Most of the reported styrene monooxygenases are involved in degrading styrene, and their activity on indole can be regarded as a the result of a relaxed substrate specificity (O’Connor et al. 1997; Panke et al. 1998; van Hellemond et al. 2007). In fact, it was shown that indole is indeed converted into the corresponding epoxide before indoxyl and indigo is formed (Heine et al. 2019) (route C in Fig. 2). Furthermore, based on sequence analysis and biochemical data, “styrene monooxygenases” can be dissected into two groups: styrene monooxygenases and indole monooxygenases (Heine et al. 2018). While the true styrene monooxygenases act on styrene, the indole monooxygenases have evolved to take part in indole degradation. As these indole monooxygenases have only been recognized very recently, more studies are needed to reveal their potential as biocatalysts for indigo production. Some styrene monooxygenases have already been explored for producing indigo. A recent study aimed at optimizing indigo production by expressing styrene monooxygenase in *Pseudomonas putida*. This resulted in 52 mg of indigo per liter of culture (Cheng et al. 2016). Progress in using styrene or indole monooxygenases for the production of indigo may benefit from previous efforts to optimize the fermentative production of (*S*)-styrene oxide using styrene monooxygenase (Park et al. 2006; Kuhn et al. 2012).

### 2-Hydroxybiphenyl 3-monooxygenase

Another FAD-dependent monooxygenase, for which indigo formation has been shown, is a laboratory-evolved mutant of 2-hydroxybiphenyl 3-monooxygenase (EC 1.14.13.44) (Meyer et al. 2002). This flavoprotein monooxygenase does not depend on a reductase; it is a single component monooxygenases which is fuelled by NADPH, belonging to the class A flavoprotein monooxygenases (van Berkel et al. 2006). During a directed evolution campaign, a mutant enzyme was identified that rendered the respective culture a dark blue color. For this engineered flavoprotein monooxygenase, it was found that indigo formation was preceded by hydroxylation of indole. Except for 3-hydroxylation of indole, resulting in indoxyl, some 2-hydroxylation also occurred, resulting in 2-oxindole. As a result, formation of some indirubin was also observed. Though a large number of class A flavoprotein monooxygenases have been reported, all acting on aromatic substrates, so far only the engineered 2-hydroxybiphenyl 3-hydroxylase was reported to act on indole to produce indigoids. As a result, relatively little data is available on such indigo-forming hydroxylases. Still, the engineered FAD-containing hydroxylase was shown to be quite productive when using recombinant *E. coli* cells, outperforming the indigo production of *E. coli* expressing naphthalene dioxygenase (Ensley et al. 1983; Meyer et al. 2002).

### Flavin-containing monooxygenases and Baeyer-Villiger monooxygenases

Another major class of FAD-containing monooxygenases, the class B flavoprotein monooxygenases (EC 1.14.13.X), has been shown to be rather rich in enzymes capable of oxidizing indole. The most prominent examples are bacterial flavin-containing monooxygenases (FMOs) (Choi et al. 2003; Singh et al. 2010; Ameria et al. 2015; Lončar et al. 2019). The first FMO efficient in indigo production was described in 2003 and was obtained from a marine bacterium, *Methylophaga aminisulfidivorans* (Choi et al. 2003). Biochemical characterization of this bacterial FMO showed that it is a dimeric and soluble enzyme which relies on NADPH and dioxygen for activity (Alfieri et al. 2008). While the enzyme is most efficient with small aliphatic amines and sulphides, it also accepts indole as substrate. It is also capable to convert indole derivatives into indigoid dyes (Rioz-Martínez et al. 2011). Through optimizing a fermentation process, using *E. coli* as recombinant expression host, an impressive indigo yield of 911 mg/L was achieved (Han et al. 2011). In the past few years, some other sequence-related indigo-producing bacterial FMOs have also been described (Ameria et al. 2015; Kim et al. 2017; Lončar et al. 2019); for the FMO from *Corynebacterium glutamicum*, high yields of indigo when using recombinant *E. coli* cells were reported (685 mg/L). The studied FMOs display exceptional high indigo productivities, making them attractive biocatalysts for the biotechnological production of this vat dye. Except for focusing on the production of indigo, a recent study has shown that FMOs can also be used for the production of indican. Coexpression of bacterial FMO together with a glucosyltransferase from the indigo plant *Polygonum tinctorium* was established in *E. coli* which resulted in the production of indican (2.9 g/L) (Hsu et al. 2018). This protected form of indoxyl represents a convenient precursor of indigo.

A subclass of class B flavoprotein monooxygenases are the type I Baeyer-Villiger monooxygenases. These FAD-containing and NADPH-dependent monooxygenases have similar structural features that also translate into a similar catalytic mechanism when compared with FMOs. However, Baeyer-Villiger monooxygenases (BVMOs) have some specific features in their active sites that allow them to perform Baeyer-Villiger oxidations (Orru et al. 2011). Yet, they also were shown to be capable to catalyze other oxidation reactions, such as sulfoxidations, epoxidations, and N-oxidations. Though no wild-type BVMO has been shown to be capable of converting indole into indigo, an engineered BVMO was discovered that could be used for indigo production, the M446G phenylacetone monooxygenase mutant (Pazmiño et al. 2007). The mutant was prepared in a successful attempt to broaden the substrate scope of this bacterial and thermostable BVMO.

### Alternative two-component flavoprotein monooxygenases

Studies have appeared in literature that indicate that also another group of flavoprotein monooxygenases can produce indigo, various class D flavoprotein monooxygenases were shown to be involved in indigo formation (Hart et al. 1990; Kosono et al. 1997; Drewlo et al. 2001; Choi et al. 2004; Alemayehu et al. 2004; Lim et al. 2005; Leveau and Gerards 2008; Lin et al. 2012; Sadauskas et al. 2017; Dai et al. 2019). All these reports involve sequence-related two-component systems in which a flavin reductase generates the reduced flavin cofactor which is subsequently used by the indole oxygenase component. No detailed studies have been carried out on these indole oxygenases. Based on sequence homology, they probably operate similar to the FAD-utilizing 4-hydroxyphenylacetate 3-hydroxylase (Thotsaporn et al. 2011). The cofactor requirement of class D flavoprotein monooxygenases (Heine et al. 2018) is variable (they are either FMN- or FAD-dependent) and still has to be determined for the described indole oxygenases. Most of these indole oxygenases have been studied in view of their role in bacterial indole degradation. Only in a recent study the potential of using such a two-component system for indigo production was demonstrated. Expression of the indole oxygenase system from *Cupriavidis* sp. SHE in *E. coli* and optimization of the medium for indigo production yielded in 307 mg indigo per liter of culture broth (Dai et al. 2019).

## Conclusions and perspectives

This review provides an overview of all enzyme types that have been shown to be able to produce indigo. After several decades of research on microbial or enzymatic indigo production, it becomes clear that a variety of redox enzymes can oxidize indole into indigo. It depends on the type of enzyme what initial oxidized product is formed, and whether side products are formed. While a number of indigo-forming oxygenases are part of a multi-component system, self-sufficient oxygenases have also been discovered or engineered. Reported productivities for indigo vary greatly and may hint to enzymes that are more suitable for indigo production. Yet, the set-up and conditions for indigo production vary enormously among studies which makes a fair comparison difficult. Future studies will resolve which enzyme system(s) are best suited for a biotechnological production of indigo.
